# A rare association between angioid streaks and pattern dystrophy

**DOI:** 10.5935/0004-2749.2022-0121

**Published:** 2023-04-10

**Authors:** Renato Bezerra Kitahara, Fernando Henrique Flores Teixeira, Fernando Moreira dos Santos, Flavio Mac Cord Medina, Mário Martins dos Santos Motta

**Affiliations:** 1 Departamento de Oftalmologia, Hospital Federal dos Servidores do Estado do Rio de Janeiro, Rio de Janeiro, RJ, Brazil

Dear Editor,

Angioid streaks (AS) are breaks that occur in a calcified and weakened Bruch’s membrane^(1,2)^. Approximately 50% of the cases are idiopathic, whereas other cases present with an associated systemic conditions, most frequently pseudoxanthoma elasticum, Paget’s disease, hemoglobinopathies, and Ehlers-Danlos syndrome^(1,2)^. Generally, they emerge between the second and fifth decades of life, depending on the associated systemic diseases^(2)^. Fundoscopic examination identifies AS bilaterally as reddish-brown, jagged, subretinal lines, with variable caliber, which radiate from the peripapillary region toward the peripheral retina. AS become darker as they develop and may increase in number, diameter, and extent^(1,2)^. Initially, AS are usually asymptomatic. Visual impairment occurs because of foveal involvement or with choroidal neovascularization (CNV)^(1,2)^.

By contrast, pattern dystrophies (PD) are a heterogeneous group of disorders that present changes in the pigment epithelium of the macular region^(3,4)^. Gass divided PD into five forms according to the pigment distribution pattern, namely, adult-onset foveomacular vitelliform dystrophy, butterfly pattern dystrophy, multifocal pattern dystrophy simulating fundus flavimaculatus, reticular dystrophy of the pigment epithelium, and fundus pulverulentus^(3,4)^. Occasionally, different patterns can coexist in the same individual. In some cases, transformations occur, and one pattern develops into another^(4)^. This case report described a case of PD-associated AS.

**Figure 1:**
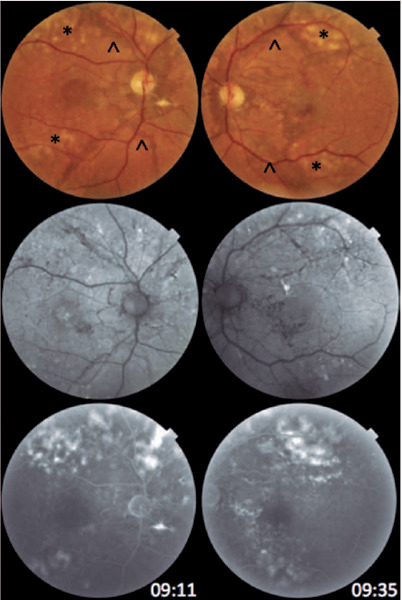
Retinography: presence of linear, jagged, darkened, and deep lesions in both eyes, starting from the optic disc toward the periphery (arrows). Yellowish, round, and confluent lesions at the posterior pole, suggestive of drusen, were also detected (*). Autofluorescence: hypoautofluorescent lines in both eyes starting from the optic disc and small, sparse hyperautofluorescent lesions at the posterior pole. Fluorescent angiography: a late image demonstrates the absence of macular leakage in both eyes, excluding the diagnosis of subretinal neovascular membrane. Areas of hyperfluorescence near the temporal arcades are visualized bilaterally.

A 61-year-old man with hypertension and a pituitary adenoma diagnosed 3 years ago visited the ophthalmology outpatient clinic for a routine appointment. He had no systemic or ophthalmologic complaints. However, his corrected visual acuity was 20/80 in the right eye and 20/40 in the left eye. The right and left intraocular pressures were 13 and 15 mmHg, respectively. No changes in the anterior segment were noted. Fundoscopic examination showed linear, darkened, and deep lesions in both eyes, starting from the optic disc toward the periphery, compatible with the diagnosis of AS. He also presented yellowish, round, and confluent lesions at the posterior pole, suggesting the appearance of multifocal vitelliform lesions (Figure 1).

**Figure 2:**
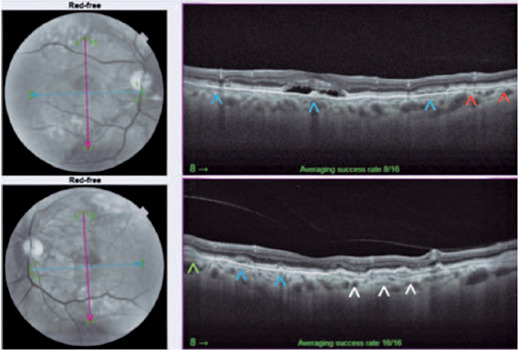
Optical coherence tomography of the right eye detected a subfoveal hyporeflective space with hyperreflective vitelliform material inside and at other points of the macula (blue arrows) and rupture of Bruch’s membrane (red arrows). In the left eye, there is a double-layer sign (white arrows), hyperreflective vitelliform material (blue arrows), and drusenoid pigment epithelial detachment (green arrow). No signs of exudation were noted.

Fundus autofluorescence (FAF) showed hypoautofluorescent lines starting from the optic disc in both eyes and small, sparse hyperautofluorescent lesions in the posterior pole (Figure 1). Optical coherence tomography (OCT) of the right eye detected a subfoveal hyporeflective space containing hyperreflective materials. In the left eye, a double-layer sign was seen, but without signs of exudation (Figure 2).

OCT angiography (OCT-A) and fundus fluorescein angiography (FFA) were performed in both eyes. No flow or leakage compatible with the diagnosis of subretinal neovascular membrane was detected. Areas of hyperfluorescence near the temporal arcades were visualized bilaterally (Figure 1).

Multimodal evaluation of the posterior segment confirmed that the case was an association of two ophthalmological diseases, AS and adult-onset foveomacular vitelliform dystrophy of the multifocal type. The concomitant presence of AS and PD has been established in the literature and commonly described in patients with pseudoxanthoma elasticum^(1)^. This diagnosis was excluded for our patient by the medical clinic and dermatology teams. This PD subtype presents with central or paracentral subretinal deposits, yellowish and round, which appear as hyperautofluorescent lesions in the FAF and hyperreflective lesions between the retinal pigment epithelium (RPE) and inner segment/outer segment (IS/OS) layer of photoreceptors in the OCT^(3,4)^. Druses, large or small, are an important differential diagnosis of vitelliform lesions, but they are located between the RPE and Bruch’s membrane^(4,5)^.

PD usually shows a slow and progressive course. A slight decrease in visual acuity and mild metamorphopsia which start around the fifth decade are the most common complaints. However, severe visual impairment can occur in up to 50% of patients after age 70 years because of geographic macular atrophy or CNV^(4)^.

The patient had two entities that were potentially progressing to CNV. In the OCT of the right eye, changes observed may lead to the erroneous diagnosis of active CNV. Through multimodal analysis using OCT-A and FFA, the changes indicated a resorbing vitelliform deposit. Owing to the possible emergence of a neovascular membrane, the patient was followed with serial tests.
